# Anthelmintic resistance in gastrointestinal nematodes of alpacas (*Vicugna pacos*) in Australia

**DOI:** 10.1186/s13071-018-2949-7

**Published:** 2018-07-04

**Authors:** Mohammed H. Rashid, Jane L. Vaughan, Mark A. Stevenson, Angus J. D. Campbell, Ian Beveridge, Abdul Jabbar

**Affiliations:** 1 Department of Veterinary Biosciences, Melbourne Veterinary School, The University of Melbourne, Werribee, Victoria 3030 Australia; 2 Cria Genesis, PO Box 406, Ocean Grove, Victoria 3226 Australia

**Keywords:** Anthelmintic, Resistance, Alpacas, Nematodes, Australia

## Abstract

**Background:**

Gastrointestinal nematodes (GINs) can cause significant economic losses in alpacas due to lowered production of fibre and meat. Although no anthelmintics are registered for use in alpacas, various classes of anthelmintics are frequently used to control parasitic gastroenteritis in alpacas in Australia and other countries. Very little is known about the current worm control practices as well as the efficacy of anthelmintics used against common GINs of alpacas. This study aimed to assess the existing worm control practices used by Australian alpaca farmers and to quantify the efficacy of commonly used anthelmintics against GINs of alpacas.

**Methods:**

An online questionnaire survey was conducted to assess current worm control practices on 97 Australian alpaca farms, with an emphasis on the use of anthelmintics. Of this group of 97 alpaca farms, 20 were selected to assess the efficacy of eight anthelmintics and/or their combinations (closantel, fenbendazole ivermectin, monepantel, moxidectin and a combination of levamisole, closantel, albendazole, abamectin) using the faecal egg count reduction test (FECRT). A multiplexed-tandem PCR (MT-PCR) was used to identify the prevalent nematode genera/species.

**Results:**

The response rate for the questionnaire was 94% (91/97). Almost half of the respondents kept alpacas with sheep and cattle, and 26% of respondents allowed alpacas to co-graze with these ruminants. Although only 63% respondents perceived worms to be an important health concern for alpacas, the majority of respondents (89%) used anthelmintics to control GINs of alpacas. The commonly used anthelmintics were macrocyclic lactones, monepantel, benzimidazoles, levamisole, closantel and their combinations, and they were typically administered at the dose rate recommended for sheep. The FECRT results showed that a combination of levamisole, closantel, albendazole and abamectin was the most effective dewormer followed by single drugs, including monepantel, moxidectin, closantel, fenbendazole and ivermectin. *Haemonchus* spp. were the most commonly resistant nematodes followed by *Trichostrongylus* spp., *Camelostrongylus mentulatus*, *Ostertagia ostertagi* and *Cooperia* spp.

**Conclusions:**

This is the first study aimed at assessing worm control practices and efficacy of commonly used anthelmintics in alpacas in Australia. Our findings document the extent of anthelmintics resistance on Australian alpaca farms and identify those anthelmintics that are still effective against GINs of alpacas.

**Electronic supplementary material:**

The online version of this article (10.1186/s13071-018-2949-7) contains supplementary material, which is available to authorized users.

## Background

In the last three decades, the farming of domesticated South American camelids (SACs), alpacas (*Vicugna pacos*) and llamas (*Lama glama*) has increased in Australia, Europe, New Zealand, the UK and the USA, due to their high-quality fibre and adaptability to many climatic conditions [1, 2]. In an intensive farming system, alpacas and llamas can be infected with both shared (those common in domestic ruminants; e.g. *Haemonchus contortus*, *Ostertagia ostertagi*, *Trichostrongylus* spp. and *Nematodirus* spp.) as well as host-specific (e.g. *Lamanema chavezi*) gastrointestinal nematodes (GINs) [3–5] that can cause significant clinical and subclinical problems, resulting in economic losses from lowered production of fibre, meat and/or leather [3, 4, 6–8]. Outside South America, knowledge on the parasites of SACs is limited.

Traditionally, the use of chemotherapeutic agents has been the most commonly used method to treat and control GINs of domestic ruminants. Similarly, farmers regularly use various classes of anthelmintics to control GINs in alpacas and llamas [3, 4], although no anthelmintic is registered for use against GINs in SACs in Australia. Given that very little is known about the pharmacokinetics of drugs in SACs [9], the off-label use of anthelmintics in alpacas registered for domestic ruminants at different dose rates recommended for goats, sheep and cattle is commonplace. However, the dose rate(s) and route(s) of administration recommended for sheep might not be effective against GINs in SACs [10] as found previously in goats [11]. Thus, under-dosing of anthelmintics may promote the development of anthelmintic resistance (AR) in GINs in SACs as under-dosing is known to be one of the risk factors for the development of AR in GINs of sheep [12]. Case reports of AR in GINs of SACs have been reported from Australia [2], Belgium [13], Canada [14] and the USA [15] in various GINs against two commonly used classes of anthelmintics, benzimidazoles and macrocyclic lactones.

Australia has the largest alpaca population (> 450,000) outside South America [16] and the Australian alpaca industry is an important emerging livestock industry. However, very little is known about the epidemiology and control of GINs in alpacas in Australia. Recently, the first case of ivermectin resistance in the Barber’s pole worm (*Haemonchus contortus*) was reported [2]. A survey of the worm control practices used by Australian alpaca farmers revealed that the dose of anthelmintics used for alpacas (e.g. one to three times of dose recommended for sheep) and the existence of other potential risk factors for the development of AR known for GINs of sheep, goats and cattle, could lead to the development of AR in GINs of alpacas (Rashid et al, unpublished data).

The aims of this study were (i) to undertake a questionnaire survey to obtain insights into farm-level characteristics that might be associated with the development of AR in GINs of alpacas and (ii) to quantify the efficacy of commonly used anthelmintics against GINs of alpacas in Australia.

## Methods

### Study population

Australia has various climatic zones, and alpaca farming in Australia mainly occurs in four zones, the Mediterranean, non-seasonal rainfall, summer rainfall and winter rainfall zones. Most alpaca herds are located in the south-eastern states of New South Wales and Victoria, with fewer in Queensland, Western Australia, South Australia and Tasmania. The majority of alpaca farms contain ≤ 50 animals, which graze year-round on pastures, with variable provision of supplementary feed (Rashid et al; unpublished data). Alpacas are routinely vaccinated against clostridial diseases (caused by *Clostridium perfringens* type D, *C. tetani*, *C. novyi* type B, *C. septicum* and *C. chauvoei*). They are generally shorn once annually in spring, although at variable times throughout the year. Timing and duration of the birthing periods vary between farms but often occur during about two months in spring. Crias are weaned at an average age of three months.

### Questionnaire survey

The survey aimed to assess current worm control practices of Australian alpaca farmers, with an emphasis on the use of anthelmintics. A questionnaire was conducted using an online programme, Research Electronic Data Capture [17]. The questionnaire contained 30 questions about (i) farm demography and general husbandry practices; (ii) the use of anthelmintics; and (iii) grazing management. The majority of questions were close-ended, with a few semi-open (i.e. a close-ended question with the addition of a category “other”). An online questionnaire survey was supplied to 97 alpaca farmers who had responded to a larger survey on more general aspects of alpaca husbandry, worm problems and parasite management in Australia (Rashid et al; unpublished data).

### Selection of farms

**Fig. 1:**
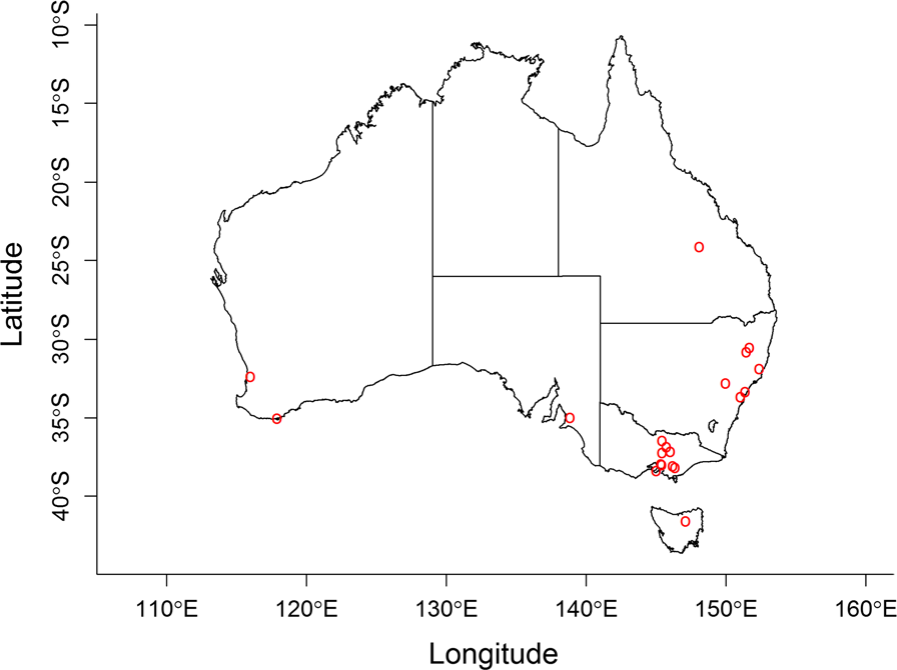
Map of Australia showing the locations of alpaca farms enrolled in the faecal egg count reduction trials in this study. Each circle represents one alpaca farm

Out of 91 alpaca farms that responded to the survey, 20 farms were selected to take part in faecal egg count reduction testing (FECRT) based on herd size and the geographical location of their herd (Fig. 1). The following selection criteria were used: (i) the herd was comprised of between 40 and 60 alpacas of different ages and sexes; (ii) deworming had not been carried out within the 8 weeks prior to the scheduled herd visit; (iii) confirmation that average faecal egg counts (FEC) were greater than or equal to 150 eggs per gram (epg) of faeces; and (iv) there was a history of anthelmintic usage on the farm in the last five years. When a farmer agreed to participate and met the first two criteria, faecal samples were collected from fifteen randomly selected alpacas and tested for FEC. Over 50 farms were tested to obtain 20 suitable farms for the FECRT trial.

### Faecal egg count reduction test (FECRT)

The FECRT was performed on each farm according to the World Association for the Advancement of Veterinary Parasitology (WAAVP) guidelines for the evaluation of anthelmintic efficacy in ruminants [18, 19]. Both female and male - Huacaya and Suri alpacas, aged 3-months to 16-years were randomly selected on each farm and allocated to five or six groups (anthelmintic treatment groups and an untreated control group) comprising 5–15 animals. Six anthelmintics were evaluated in this study: (i) monepantel (Zolvix®, Elanco Pty. Ltd., West Ryde, New South Wales, Australia); (ii) a combination of levamisole, closantel, albendazole and abamectin (Q-drench®, Jurox Pty. Ltd., Rutherford, New South Wales, Australia); (iii) closantel with sodium selenate (Closicare Plus Selenium®, Virbac Pty. Ltd., Milperra, New South Wales, Australia); (iv) ivermectin (Ivomec®, Boehringer Ingelheim Pty. Ltd., North Ryde, New South Wales, Australia); (v) moxidectin (Cydectin® Injection for cattle, Virbac, Pty. Ltd., Milperra, New South Wales, Australia); and (vi) fenbendazole (Panacur 25®, Intervet Pty. Ltd., Bendigo East, Victoria, Australia). Resistance to ivermectin was found up to the 9th FECR trial. Hence, we decided to replace ivermectin with a more potent macrocyclic lactone in subsequent FECR trials. All anthelmintics were administered orally apart from moxidectin (subcutaneously) at 1.5 times the dose rate recommended for sheep. Animals were dosed individually based on body weight using scales where available.

Individual faecal samples were collected from the rectum pre- (day 0) and post-treatment (day 11–14) into zip-lock plastic bags, and were kept at 4 °C until processed for FEC using a modified McMaster method [20] within seven days of collection. Briefly, four grams of faeces were mixed with 11 ml of water added into a 60 ml container and soaked for 5–30 min before making a homogenized faecal slurry. Saturated sugar (specific gravity 1.27) solution (45 ml) was added and 30–45 min later, the sample was agitated, and a sample drawn immediately from the suspension using a sieve-top pipette (sieve aperture size 12 meshes per cm), to fill two chambers of a Whitlock egg counting slide (http://www.whitlock.com.au/slides/JAWCO_Home.htm). After five minutes, the slide was placed on the stage of a compound light microscope and eggs were counted. The sensitivity of the McMaster technique was 15 EPG.

### Nematode identification

We used a newly established molecular diagnostic kit (Easy-Plex, AusDiagnostics Pty. Ltd., Beaconsfield, Australia) for the identification of common GINs of alpacas [21]. Faecal DNA was extracted using a method described previously with few modifications [22]. Pre- and post-treatment faecal samples for each group were pooled in order to obtain one DNA sample representing 5–15 individual faecal samples per group. This was achieved during processing of individual faecal samples for FEC by withdrawing 1 ml of the faeces suspension in the saturated sugar solution to a 50 ml Falcon tube. This process was repeated for each sample per treatment group. Finally, the Falcon tube was filled by adding more floatation solution to make 50 ml. Following centrifugation (2500× *rpm*, 10 min), the supernatant (~5 ml) was collected and transferred to another 50 ml Falcon tube. To wash the GIN eggs collected in the Falcon tube, tap water was added to 50 ml and centrifuged (2500× *rpm*, 10 min). The supernatant was discarded and the pellet was washed twice more, using the same steps as above. The washed pooled eggs with remaining faecal materials were transferred into a microcentrifuge tube and stored at -20 °C until further use. Following thawing, a 250 μl sample of the concentrated faecal material was used to extract and isolate DNA using Powersoil® DNA purification kit (MoBio, USA) as per manufacturer’s protocol.

The assay was conducted in the *High*-Plex 24 system with the MT-Assay Setup Software for the first round of PCR and the 96-well MT-Analyzer and the MT Analysis Software (Cat. No. 9150, AusDiagnostics, Mascot, New South Wales, Australia) for the second round of PCR. The primary amplification (‘target enrichment’) was conducted using nematode-specific primer pairs designed for the sequences of the Internal Transcribed Spacer 2 (ITS 2) [Step 1 tubes for nematodes (8-well), Cat. No. 78150S, AusDiagnostics]. The secondary amplification for semi-quantification employed nested primer pairs to the internal regions of the ITS 2 (Alpaca Nematodes MP96 8-well, Cat. No. 78150E, AusDiagnostics) specific to *Camelostrongylus mentulatus*, *Cooperia* spp., *Haemonchus* spp., *Ostertagia ostertagi*, *Oesophagostomum* spp., *Teladorsagia circumcincta* and *Trichostrongylus* spp. These internal primer pairs amplify a region of ~90 to 110 bp from the ITS 2 region. Furthermore, another primer pair was included in each run as a reference to assess the efficiency of amplification from 10,000 copies of a synthetic oligonucleotide template (internal ‘spike control’).

For primary amplification (15 cycles of 10 s at 95 °C, 20 s at 60 °C, and 20 s at 72 °C), 5 μl of genomic DNA representing each DNA sample or 5 μl of water (negative control) were dispensed into 0.2 ml PCR strips and placed into a 24-well thermocycling block in the *High*-Plex 24 system (AusDiagnostics). Subsequently, the analysis was executed by the program MT-Assay Setup Software (AusDiagnostics). Following the first round of PCR, the secondary amplification and the melting curve analysis were performed in a 96-well MT-Analyzer using the MT Analysis Software (AusDiagnostics). Each sample was recorded as test-positive using the auto-call function of the Easy-Plex software (AusDiagnostics) if the amplicon produced a single melting curve which was within 1.5 °C of the expected melting temperature, the height of the peak was higher than 0.2 dF/dT (where dF/dT is the derivative of fluorescence over temperature), and the peak width was ≤ 3.5 °C, otherwise the sample was considered as negative (AusDiagnostics). Additionally, cycle threshold (Ct) values for each nematode per sample were determined by comparing with the data obtained from the internal spike control which had a known 10,000 DNA copy numbers. Based on the peak high-resolution melting (HRM) temperature analyses, nematode genera/species were assigned according to their mean HRM temperatures. Randomly selected amplicons representing each nematode genus/species were subjected to sequencing to verify the target nematodes.

### Statistical analyses

Questionnaire data were downloaded from REDCap as a comma-separated values (CSV) file. Data validation and cleaning were performed by using Microsoft Excel 2013. Calculation of faecal egg count reduction (FECR) was performed using the contributed R package “e*ggsCount*” [23] and following the WAAVP guidelines [19, 24]. FECR (%) was calculated between treatment and control group at post-treatment collection for each anthelmintic. Faecal egg count data are generally over-dispersed and inherit Poisson errors, which was incorporated in the R package “*eggsCount*” [24]. Thus, it has advantages over Excel spreadsheets to calculate FECR more precisely by taking the inherent errors of faecal egg count data into account.

### Interpretation of the FECRT results

Anthelmintic resistance status was interpreted as recommended by the WAAVP guidelines on AR based on the percentage of faecal egg count reduction (%FECR) and the upper (UCL) and lower (LCL) 95% confidence limits [18]. Hence, each anthelmintic was declared as (i) effective when the %FECR and UCL were both ≥ 95% and the LCL was ≥ 90%, (ii) suspected resistant when %FECR was < 95% or LCL was < 90%, and (iii) ineffective/resistant when both %FECR was < 95% and LCL was < 90%. Furthermore, multiple AR was declared when parasite populations of GINs were identified, using the above criteria, to be resistant to anthelmintics of different chemical classes [25].

## Results

### Questionnaire survey

The response rate for the questionnaire was 94% (91/97). Huacaya was the more popular alpaca breed, with an average herd size of 77 (minimum 9; maximum 600) alpacas. About half of the respondents (51%, 46/91) kept alpacas with other livestock species such as sheep and cattle, and 26% (24/91) of alpaca farmers allowed their animals to co-graze with other domestic ruminants (Table 1). Twenty five percent (23/91) of respondents had agisted non-home-bred alpacas on their farms at least once during the last five years. Although 63% (57/91) of respondents reported that worms were an important health issue for their alpacas, the majority of respondents (89%, 81/91) used anthelmintics for the control of GINs in their animals (Table 1). The commonly used anthelmintics were macrocyclic lactones (e.g. ivermectin, moxidectin; 65% (53/81)), monepantel (31%, 25/81), benzimidazoles (BZs) (20%, 16/81), levamisole (LEV) (15%, 12/81), closantel (10%, 8/81), and their combinations, including two [BZ and MLs, 7% (6/81)], three [BZ, LEV and MLs, 9% (7/81)] or four [closantel, BZ, LEV and MLs, 37% (29/81)] anthelmintics (Table 1). The majority of respondents (53%, 43/81) used these anthelmintics at the dose rate recommended for sheep, though some used 1.5 times the dose rate recommended for sheep (23%, 19/81) or the dose rate recommended for cattle (10%, 8/81). The use of anthelmintics seemed to be need-based (62%, 50/81) rather than a part of any strategic deworming program twice (25%, 20/81) or once per year (12%, 10/81). Alpacas were dewormed either before winter (43%, 35/81), at shearing (36%, 29/81) or at weaning (32%, 26/81) (Table 1). The majority of respondents (60%, 49/81) were using one class of anthelmintics for more than a year, and most of them (72%, 58/81) said that they changed (‘rotated’) drench classes in the last five years. A relatively small proportion of respondents (12%, 11/91) reported that they tested the efficacy of anthelmintics in use on their farms using FECRT (Table 1).

**Table 1:** Worm control practices used by Australian alpaca farms included in this study

Worm control factor	No. of responses	Responses (%)
Keeping alpacas with other livestock species	46/91	51
Co-grazing of alpacas with other livestock species	24/91	26
Keeping agisted alpacas	23/91	25
Worms is an important health issue of alpacas	57/91	63
Anthelmintic usage	81/91	89
Types of anthelmintics used		
Macrocyclic lactones (MLs)	53/81	65
Monepantel	25/81	31
Benzimidazole (BZ)	16/81	20
Levamisole (LEV)	12/81	15
Closantel	8/81	10
Double combinations (BZ and LEV)	6/81	7
Triple combinations (BZ, LEV and ML)	7/81	9
Q-Drench (a combination of levamisole, closantel, albendazole, abamectin)	29/81	37
Deworming time		
Before winter	35/81	43
At shearing	29/81	36
At weaning	26/81	32
Dose rate		
Sheep dose	43/81	53
1.5 times sheep dose	19/81	23
Cattle dose	8/81	10
Testing of anthelmintics for efficacy (e.g. FECRT)^a^	11/91	12
Rotation of anthelmintics	58/81	72

^a^*FECRT* faecal egg count reduction test

### Faecal egg count reduction tests

**Fig. 2:**
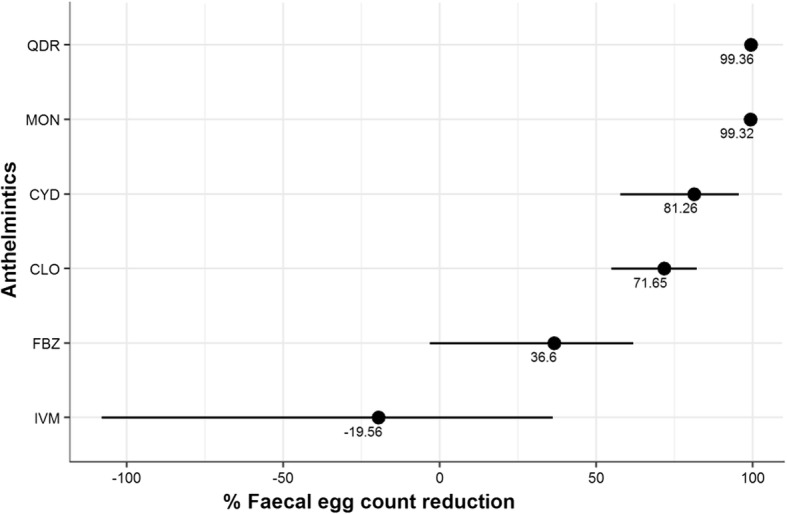
Overall efficacy of six anthelmintics against gastrointestinal nematodes of alpacas on 20 farms in Australia. Each circle shows percentage of the faecal egg count reduction while each horizontal line shows upper and lower 95% confidence intervals. *Abbreviations*: CLO, closantel; CYD, cydectin; IVM, ivermectin; FBZ, fenbendazole; QDR, Q-drench (a combination of abamectin, albendazole, closantel and levamisole); MON, monepantel

**Fig. 3:**
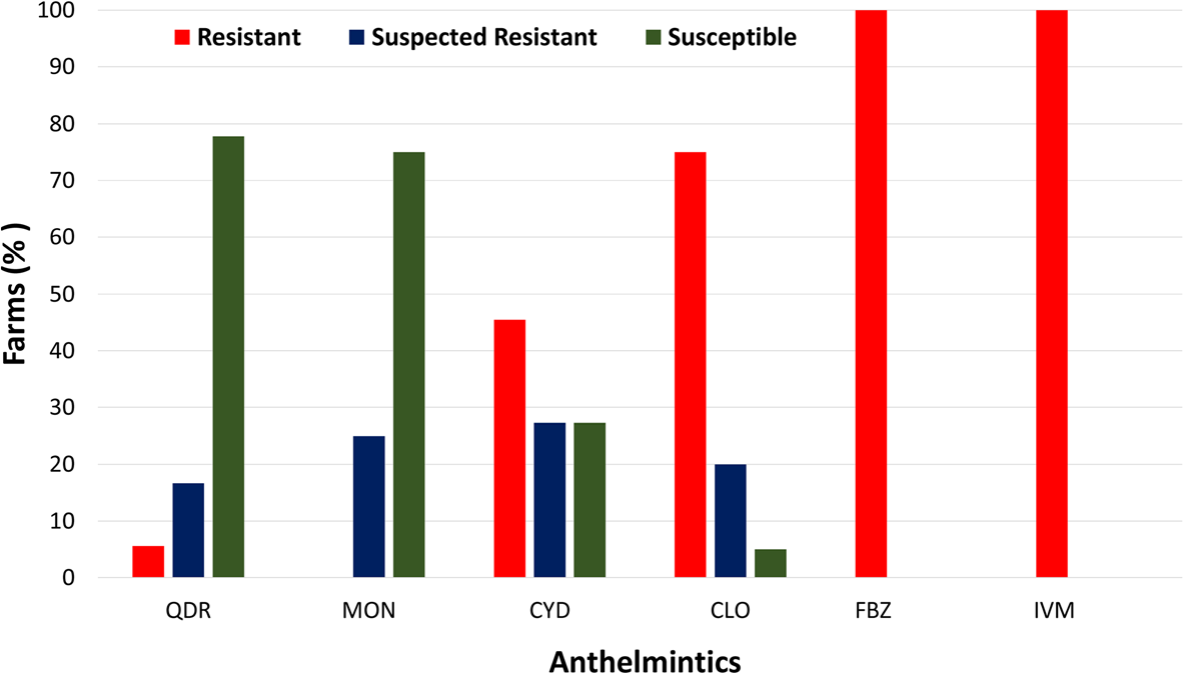
The proportion of farms with resistance, suspected resistance and susceptibility of gastrointestinal nematodes of alpacas to six anthelmintics on 20 farms in Australia. *Abbreviations*: CLO, closantel; CYD, cydectin; IVM, ivermectin; FBZ, fenbendazole; QDR, Q-drench (a combination of abamectin, albendazole, closantel and levamisole); MON, monepantel

**Fig. 4:**
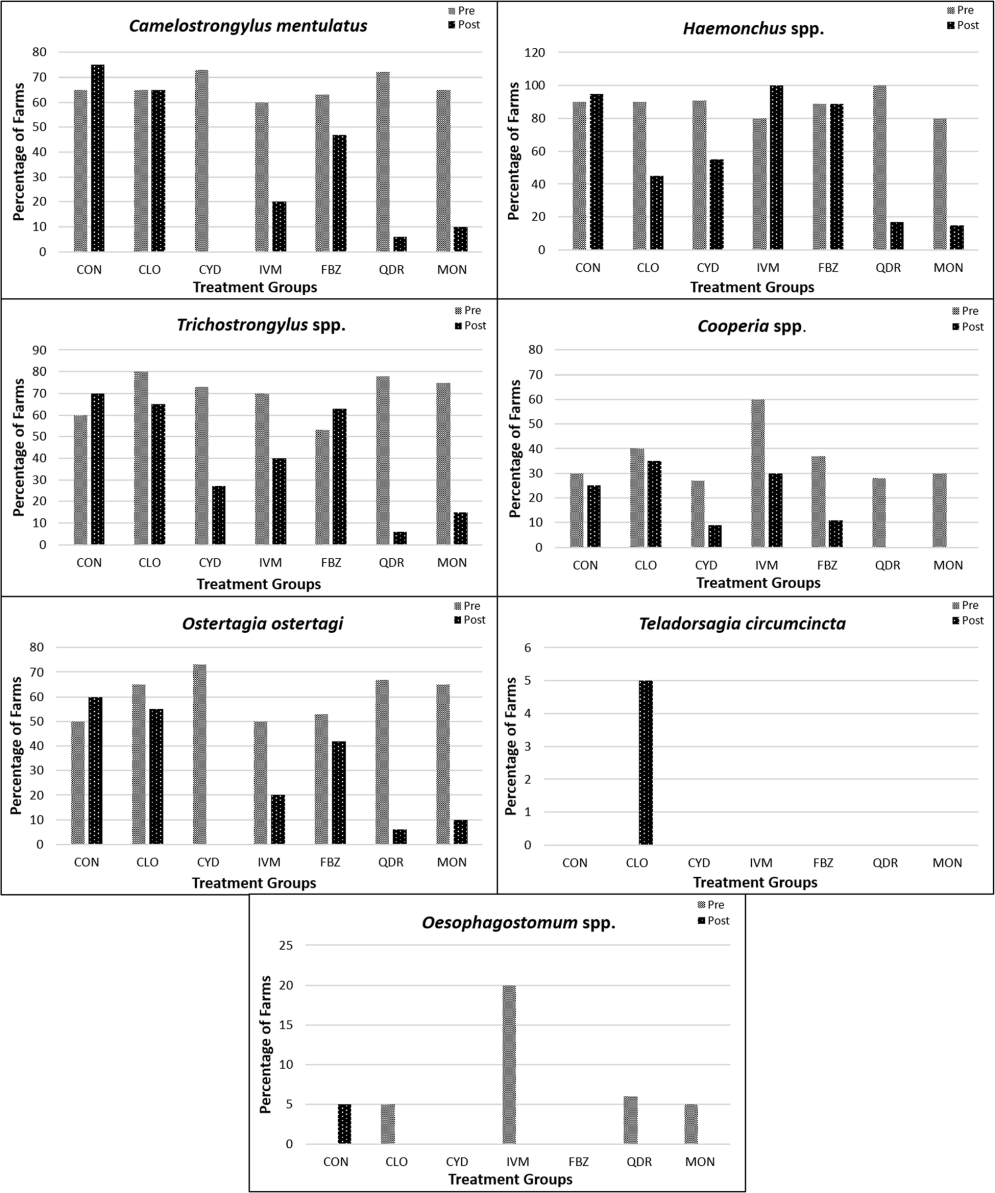
Gastrointestinal nematodes found in faeces of alpacas pre- and post-treatment with six anthelmintics on alpaca farms in Australia. The nematodes were identified using the multiplexed-tandem PCR. *Abbreviations*: CLO, closantel; CON, negative control; CYD, cydectin; IVM, ivermectin; FBZ, fenbendazole; QDR, Q-drench (a combination of abamectin, albendazole, closantel and levamisole); MON, monepantel

**Fig. 5:**
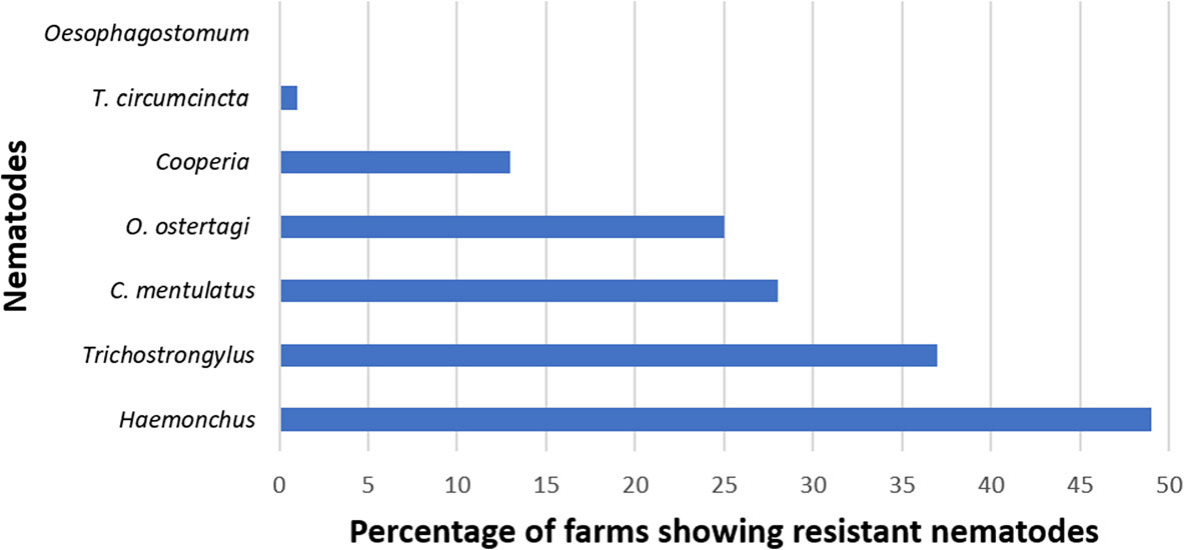
Percentage of resistant gastrointestinal nematodes against commonly used anthelmintics on 20 alpaca farms in Australia

The FECRT results revealed that a combination of levamisole, closantel, albendazole and abamectin was the most effective dewormer (78%, 14/18 susceptible farms), followed by monepantel (75%, 15/20), moxidectin (27%, 3/11), closantel (5%, 1/20), fenbendazole (0%, 0/19) and ivermectin (0%, 0/10) (Figs. 2 and 3; Table 2; see Additional file 1: Table S1). The molecular identification of GINs from all herds of alpacas showed the presence of mixed GIN infections containing *C. mentulatus*, *Cooperia* spp., *Haemonchus* spp., *O. ostertagi*, *Oesophagostomum* spp., *T. circumcincta* and *Trichostrongylus* spp. *Haemonchus* spp. (49%) were the most commonly resistant nematodes followed by *Trichostrongylus* spp. (37%), *C. mentulatus* (28%), *O. ostertagi* (25%) and *Cooperia* spp. (13%) whereas *Oesophagostomum* spp. (100%) and *T. circumcincta* (99%) were susceptible to anthelmintics tested in this study (Figs. 4 and 5; see Additional file 1: Table S2).

**Table 2:** Faecal egg count reduction percentages (%FECR) and 95% confidence intervals (CI) calculated by the faecal egg count reduction test (FECRT) 10–14 days after anthelmintic treatments in alpacas naturally infected with gastrointestinal nematodes on 20 alpaca farms in Australia

Farm no.	%FECR (95% CI) of anthelmintics					
	Monepantel	Q-drench^a^	Closantel	Fenbendazole	Ivermectin	Moxidectin
1	100	NT^b^	98 (93–100)	64 (-55–95)	42 (-270–91)	NT
2	98 (94–100)	NT	54 (-3–80)	-60 (-286–33)	-329 (-1157–46)	NT
3	99 (90–100)	100 (94–100)	86 (17–98)	NT	40 (-224–87)	NT
4	99 (87–100)	99 (96–100)	93 (66–98)	87 (34–97)	45 (-217–91)	NT
5	100	100	92 (72–97)	56 (-44–87)	-58 (-589–63)	NT
6	95 (75–99)	100	25 (-168–79)	44 (-250–91)	-31 (-293–56)	NT
7	98 (78–100)	100	80 (-1–96)	8 (-254–76)	21 (-269–83)	NT
8	100	100	34 (-32–67)	58 (11–80)	87 (54–97)	NT
9	100	100	-65 (-1291–80)	-522 (-4368–14)	-7 (-618–84)	NT
10	97 (75–100)	99 (92–100)	29 (-44–65)	55 (-7–81)	2 (-280–74)	39 (-355–92)
11	100	96 (67–100)	66 (10–87)	15 (-201–76)	NT	93 (65–98)
12	100	100	97 (81–99)	75 (-59–99)	NT	98 (84–100)
13	100	99 (92–100)	90 (47–98)	94 (82–98)	NT	99 (85–100)
14	100	99 (86–100)	95 (69–99)	-203 (-1396–39)	NT	98 (84–100)
15	100	100	49 (-104–87)	70 (13–90)	NT	100
16	100	93 (42–99)	96 (79–99)	50 (-174–91)	NT	1 (-566–85)
17	100	100	-194 (-1262–37)	9 (-118–62)	NT	89 (-2–99)
18	100	100	96 (48–100)	78 (-33–96)	NT	90 (23–99)
19	99 (85–100)	99 (85–100)	-13 (-327–70)	-147 (-848–36)	NT	100
20	100	100	-96 (-3257–89)	-18 (-1560–92)	NT	100

^a^Q-drench contains levamisole, closantel, albendazole, abamectin

^b^*NT* not tested

Ivermectin and moxidectin, the two compounds tested as single ML anthelmintics, were infrequently effective at the dose rates used in this study (Fig. 3; Table 2). On the 10 farms where ivermectin was tested, the FECR on six farms was 2–87% and there was no reduction in FEC on the remaining four farms (i.e. FECR -329 to -7%) (Fig. 3; Table 2). Conversely, moxidectin was fully effective on three farms but was not effective on the remaining eight farms, with three farms having suspected resistance and the other five resistant GINs (Fig. 3; Table 2). BZ used as a single anthelmintic in this study was ineffective against GINs of alpacas on all farms, with FECR ranging between 8–94% on 13 farms, with an increase in epg (FECR -522 to -18%) on six farms. Similarly, GINs were found to be susceptible to closantel on only one farm, while resistance and suspected resistance to this drug occurred on 15 (FECR of 29–93%) and four (FECR -194 to -13%) alpaca farms, respectively. Monepantel and a combination of anthelmintics were found to be the most effective drenches on alpaca farms in this study, with the majority of prevalent GINs ~100% susceptible to both anthelmintics (Fig. 3; Table 2). Multiple AR (when two or more anthelmintics from different classes are ineffective) was detected on all alpaca farms included in the study (Table 2).

Molecular detection of seven common GINs in pre- and post-treatment pooled faecal samples of alpacas revealed that monepantel and a combination of anthelmintics were most successful in eliminating all the GINs (i.e. *C. mentulatus*, *Cooperia* spp., *Haemonchus* spp., *O. ostertagi*, *Oesophagostomum* spp., *T. circumcincta* and *Trichostrongylus* spp.) prevalent on 20 alpaca farms (Fig. 4; see Additional file 1: Table S1). Fenbendazole had no effect on *Haemonchus* spp. and *Trichostrongylus* spp., with very little effect on *C. mentulatus*, *O. ostertagi* and *Cooperia* spp. Likewise, ivermectin showed no efficacy against *Haemonchus* spp. with variable effect on other GINs (Fig. 4). Moxidectin seemed to have the highest efficacy against *C. mentulatus* and *O. ostertagi* while it was moderately effective against other five GINs (Fig. 4). Closantel is a narrow-spectrum anthelmintic and known to be effective against blood feeding nematodes such as *Haemonchus* spp. However, it was not effective against this important nematode in all alpaca farms studied herein (Fig. 4).

## Discussion

Gastrointestinal nematodes are a major clinical and economic threat to SACs throughout the world. Since there are no anthelmintics registered for use in SACs, limited information is available on their efficacy and safety. This is the first study to comprehensively investigate the efficacy of commonly used anthelmintics against GINs of alpacas and llamas. The combination of levamisole, closantel, albendazole and abamectin was the most commonly effective dewormer on the 20 Australian alpaca farms in this study, followed by monepantel, moxidectin, closantel, fenbendazole and ivermectin. *Haemonchus* spp. were the most commonly resistant nematodes followed by *Trichostrongylus* spp., *C. mentulatus*, *O. ostertagi* and *Cooperia* spp. Previously, AR in GINs of alpacas and llamas had been reported from Australia [2], Belgium [13], Canada [14] and the USA [15] against two commonly used classes of anthelmintics, benzimidazoles and macrocyclic lactones in *H. contortus*. However, almost all of these studies were case reports, including the one from Australia [2] as opposed to this study that provides insights into the problem of AR in GINs of alpacas at a national level across the alpaca industry in Australia - the country which has the highest number of alpacas outside South America.

In this study, the questionnaire survey revealed invaluable information about the husbandry practices on Australian alpaca farms. Over 50% of farmers kept alpacas with sheep and cattle as well as allowing them to graze with domestic ruminants that can expose alpacas to shared GINs such as *Haemonchus* spp. [3, 5, 26–28], sometimes resulting in fatal infections [2, 6]. Similarly, keeping different livestock species that can harbour similar GINs on the same property, can increase the transmission of resistant GINs among ruminants. Previously, Edwards et al. [29] found the highest prevalence of levamisole resistant *Trichostrongylus* sp. in sheep where sheep and cattle had grazed together on the same paddock in Western Australia; however, such studies have not been undertaken for SACs. Furthermore, we found that agistment of alpacas occurred on 25% of Australian alpaca farms surveyed. This practice may further increase the risk of introducing resistant GINs to a herd if proper quarantine deworming and/or procedure(s) are not followed.

The FECRT results of this study should be interpreted carefully as we tested various anthelmintics in alpacas at 1.5 times the dose rate recommended for sheep because no information is available on the therapeutic doses of commonly used anthelmintics in SACs. Furthermore, the number of animals per group of treatment for the FECRT on some farms was less (see Table 2) than that (10–15 animals per group) recommended by the WAAVP for evaluating the efficacy of anthelmintics against GINs of ruminants [18] due to lack of the required number of animals per farm. Additionally, two MLs, ivermectin and moxidectin were not tested on all farms as we wanted to assess the efficacy of both a less potent (ivermectin) as well as a more potent (moxidectin) ML drug in alpacas. Furthermore, we could not test these two drugs on any farm together due to the lack of alpacas for the FECRT at any farm.

Fenbendazole and albendazole are widely used broad-spectrum dewormers in sheep and cattle [30]. Anthelmintic resistance to BZs was reported in GINs of sheep from Australia soon after the introduction of thiabendazole [31]. Recently, Playford et al. [32] conducted a national survey to assess the prevalence of AR in GINs of sheep in Australia and they found that 96% of farms in Australia had resistant *H. contortus*, *T. circumcincta* and *Trichostrongylus* spp. Similarly, we found that none of the 20 alpaca farms included in this study had susceptible worms (as per guidelines of the WAAVP) against fenbendazole at 7.5 mg/kg body weight, with an overall FECR of 36% (see Fig. 2, Table 2). It completely failed to reduce the number of *Haemonchus* spp. and *Trichostrongylus* spp., and was only partially successful in reducing the numbers of *C. mentulatus*, *Cooperia* spp., *O. ostertagi* and *Oesophagostomum* spp. (see Fig. 4). Previously, BZ resistance in GINs of alpacas has been reported from the USA [14] where no FECR (-111%) was observed when fenbendazole was given orally at 10 mg/kg compared with 59% when albendazole was used at the same dose rate. The second BZ used in this study was albendazole in combination with other anthlemintics, levamisole, closantel and abamectin, which proved to be one of the most effective anthelmintics against GINs of alpacas. The efficacy of BZ in this combination formulation cannot be evaluated separately as the high efficacy of this group might be due to the presence of effective anthelmintic(s) when two or more classes of anthelmintics are used [33].

In this study, ivermectin failed to reduce FECs (-20%, see Fig. 2) of nematodes when given orally at 300 μg/kg. In addition, it was unsuccessful in reducing the numbers of *Haemonchus* spp. but was partially effective against *C. mentulatus*, *Cooperia* spp., *O. ostertagi*, *Oesophagostomum* spp. and *Trichostrongylus* spp. (see Fig. 4). Similar results were found in alpacas and llamas from the USA by Gillespie et al. [15] when they used the same dose rate and the route of administration of ivermectin against *H. contortus*. However, they found better efficacy of ivermectin in llamas on two farms (FECR 22–37%) than on one alpaca farm (FECR -65%). In a previous study from Australia, Jabbar et al. [2] also found *H. contortus* in alpacas resistant to ivermectin (FECR 35%) when given orally at 200 μg/kg. Ivermectin resistance in strongyles of alpacas has also been reported from Peru [45]. The questionnaire survey results of this study revealed that MLs were the most commonly used anthelmintics by Australian alpaca farmers, and the high frequency of the use of dewormers have been found to be associated with the development of AR in sheep GINs [34]; hence, this might also be the case for the development of AR in GINs of SACs.

The second ML, moxidectin, used in this study was 81% effective against GINs of alpacas at farm level, and resistance was reported on 46% farms, with 27% farms susceptible and 27% suspected for resistance. This is the first report of moxidectin resistance against GINs in alpacas. However, Gillespie et al. [15] reported moxidectin resistance against *H. contortus* in llamas from the USA. The third ML used in this study was abamectin in combination with other anthlemintics, albendazole, levamisole and closantel which proved to be one of the effective anthelmintics against GINs of alpacas.

Closantel is a narrow spectrum anthelmintic and is recommended for *H. contortus* in small ruminants in Australia. Findings of this study showed that 75% of the 20 alpaca farms had resistant populations of GINs. However, this result should be interpreted carefully as closantel is not claimed to be effective against the majority of GINs (*C. mentulatus*, *Cooperia* spp., *O. ostertagi*, *Oesophagostomum* spp., *T. circumcincta* and *Trichostrongylus* spp.) found in alpacas on the 20 farms. Regarding its efficacy against *Haemonchus* spp., this nematode was found on 18 (out of 20) farms but closantel was effective only on 50% (9/18) farms. Similar results were found by Playford et al. [32] where they reported 43% (23/53) prevalence of closantel resistance in *H. contortus* of sheep from Australia. Conversely, Jabbar et al. [2] found that an ivermectin-resistant population of *H. contortus* in alpacas was 99% susceptible to closantel. Given that *H. contortus* can lead to fatal infections in alpacas and it is resistant to most of the commonly used anthelmintics, closantel should be used very carefully in areas/farms where its resistance has not been reported, thereby delaying the development of AR against specific, narrow spectrum anthelmintics.

This is the first report documenting the efficacy of monepantel against GINs in alpacas. We did not find monepantel resistance in GINs of alpacas when used at 3.75 mg/kg body weight. However, as per the guidelines of the WAAVP, monepantel resistance was suspected on five farms (see Fig. 2). Previously, Dadak et al. [10] undertook a study aimed at establishing an effective dose rate of monepantel for treating GINs in llamas in Austria, and found that three dose rates of 2.5 mg/kg, 5.0 mg/kg and 7.5 mg/kg of monepantel were able to reduce FECs by 84%, 93% and 100%, respectively. However, we used 1.5 times the dose rate recommended for sheep herein as it was the most commonly used dose by Australian alpaca farmers as well as veterinarians (J. Vaughan, unpublished data). In addition, we found that monepantel at this dose rate was 100% effective against GINs on 13 alpaca farms while 95–99% effective on seven farms (see Table 2). These differences in the efficacy of monepantel in two species of SACs using different doses might be associated with differences in the pharmacokinetic properties of the drug in alpacas and llamas. However, this proposal warrants further investigation. Although monepantel is a relatively new drug, GINs of sheep resistant to this drug have been reported from Australia [35], Brazil [36], New Zealand [37], Netherlands [38] and Uruguay [39]. Similarly, the injudicious and frequent use of monepantel can also lead to the development of AR in GINs of alpacas. Therefore, care should be taken when using monepantel to prolong its efficacy against GINs of alpacas as this is one of the two dewormers found to be effective herein.

This study presents multiple AR in GINs of alpacas for the first time as most of them were resistant to fenbendazole, closantel and ivermectin on different Australian alpaca farms (see Fig 4). Multiple anthelmintic resistance occurs when two or more classes of anthelmintics are unable to control GIN populations that were previously susceptible (more than 95% killed) to anthelmintics at their therapeutic doses [40]. Previously, Gillespie et al. [15] documented *H. contortus* resistance to ivermectin, fenbendazole and moxidectin in llamas from the USA. Multiple resistance to anthelmintics is very common among the main GINs that infect sheep [40], goats [41] and cattle [42]. Given that Australia is among the world leaders for the high prevalence of multiple AR in GINs of small ruminants [32], more sustainable strategies to control GINs in alpacas are required to control the problem of multiple AR.

This study utilised a newly established molecular diagnostic technique (MT-PCR) to identify nematode genus/species of alpacas in pre- and post-treatment pooled faecal DNA samples [21]. Traditionally, larval cultures (LC) are used to identify nematode genus/species. However, this procedure is time-consuming and lacks sensitivity and specificity. In addition, the LC requires experienced personnel for accurate identification of the third-stage larvae (L3s) as many nematode species are difficult to distinguish morphologically [43]. The MT-PCR assay used in this study also allowed an accurate identification of one of the common nematodes of alpacas, *C. mentulatus* [21] as LC does not allow its reliable identification due to unavailability of morphological keys of the third-stage larvae. Therefore, the testing of pre- and post-treatment pooled faecal DNA samples from 20 alpaca farms allowed us, for the first time, to ascertain the efficacy of closantel, fenbendazole, ivermectin, monepantel moxidectin and a combination of four anthelmintics against *C. mentulatus* as well as other GINs (see Fig. 4). Hence, the MT-PCR assay should be used in future epidemiological and AR studies to accurately identify the common GINs of alpacas as well as llamas.

We assessed the efficacy of a combination of four classes of anthelmintics for the first time in alpacas and it was found to be the most effective dewormer in alpacas in this study. Recently, there has been growing interest in the use of combinations of anthelmintic classes for the control of GINs of ruminants as multiple AR is an emerging threat for the control of nematode parasites [33, 44, 45]. Given that we found multiple AR for all anthelmintics when used as a single dewormer, the use of combinations of two or more anthelmintics with good efficacy as single dewormer(s) could be an effective means of delaying the development of AR in GINs of alpacas and llamas. However, future large-scale studies will be required to test a variety of combinations of anthelmintics against GINs of SACs.

Given that no anthelmintics are registered for use against GINs in SACs and very little is known about pharmacokinetic properties of the commonly used anthelmintics in alpacas and llamas, an appropriate dose rates and the route(s) of administration for various anthelmintics in these animals are unknown. For example, Guerden & Hemelrijk [46] found that ivermectin reduced 100% FECs of GINs in both alpacas and llamas when used subcutaneously at a dose rate of 200 μg/kg body weight. However, Windsor et al. [8] reported that subcutaneous administration of ivermectin reduced but did not completely eliminate GIN infections in alpacas. Conversely, the oral administration of ivermectin at a dose rate of 300 μg/kg body weight did not result in the reduction of GINs of alpacas in the USA (FECR -65%; [15]) or in Australia (FECR -20%, this study). These differences in the efficacy of ivermectin in the above four studies might be due to the different route of administration (oral *vs* subcutaneous) as the serum concentration of ivermectin was lower in llamas following its administration at a dose rate of 200 μg/kg body weight orally (less than 2 ng/ml) than subcutaneously (3 ng/ml) [17]. However, Burkholder et al. [47] were not able to find detectable levels of ivermectin in serum of llamas when ivermectin was injected subcutaneously at a dose rate of 200 μg/kg body weight. The pharmacokinetics of ivermectin have not been studied in alpacas but we cautiously expect that the serum concentration of ivermectin in alpacas after the administration *via* various routes will be very similar to those found in llamas. Such limited but conflicting reports on pharmacokinetics of anthelmintics in SACs complicate the situation and do not provide sound evidence-based practice for using an accurate dose rate and route(s) of administration for different anthelmintics in alpaca and llama medicine. Therefore, large-scale pharmacokinetic studies are needed to understand pharmacokinetic properties, appropriate dose rate(s) and the route(s) of administration of the commonly used anthelmintics in alpacas and llamas.

## Conclusions

This is the first study to comprehensively investigate the efficacy of commonly used anthelmintics against GINs of alpacas and llamas. The combination of levamisole, closantel, albendazole and abamectin was the most commonly effective dewormer on the 20 Australian alpaca farms in this study, followed by monepantel, moxidectin, closantel, fenbendazole and ivermectin. *Haemonchus* spp. were the most commonly resistant nematodes followed by *Trichostrongylus* spp., *C. mentulatus*, *O. ostertagi* and *Cooperia* spp. This study highlights the need for future large-scale pharmacokinetic studies to understand pharmacokinetic properties, appropriate dose rate(s) and the route(s) of administration of the commonly used anthelmintics in SACs.

## Additional file

Additional file 1: **Table S1.** Arithmetic means, minimum faecal egg counts (FEC; eggs per gram of faeces, EPG) and maximum FEC counts before and after treatment with different anthelmintics on 20 Australian alpaca farms. **Table S2.** Effect of different anthelmintics on the common gastrointestinal nematodes before and after treatment in naturally infected alpacas on 20 alpaca farms in Australia. (DOCX 44 kb)
