# Supplementary material for: Anthelmintic resistance in gastrointestinal nematodes of alpacas (Vicugna pacos) in Australia
Source: Parasit Vectors. 2018 Jul 4;11:388. doi: 10.1186/s13071-018-2949-7 (PMC6031175; doi:10.1186/s13071-018-2949-7)
Supplement: Supplementary file 1 — Table S1. Arithmetic means, minimum faecal egg counts (FEC; eggs per gram of faeces, EPG) and maximum FEC counts before and after treatment with different anthelmintics on 20 Australian alpaca farms. Table S2. Effect of different anthelmintics on the common gastrointestinal nematodes before and after treatment in naturally infected alpacas on 20 alpaca farms in Australia. (DOCX 44 kb) [file 13071_2018_2949_MOESM1_ESM.docx]

**Additional file 1:** **Table S1.** Arithmetic means, minimum faecal egg counts (FEC; eggs per gram of faeces, EPG) and maximum FEC counts before and after treatment with different anthelmintics on 20 Australian alpaca farms

| Farm no. | State | No. of alpacas per group | Anthelmintic | Pre-treatment FEC | | Post-treatment FEC | |
| --- | --- | --- | --- | --- | --- | --- | --- |
|  |  |  |  | EPG ± SD | Min-Max | EPG ± SD | Min-Max |
| 1 | VIC | 7 | MON | 624 ± 750 | 45-2220 | 0 | 0 |
|  |  | 7 | CLO | 799 ± 402 | 255-1275 | 9 ± 12 | 0-30 |
|  |  | 7 | FBZ | 208 ± 244 | 0-600 | 182 ± 269 | 0-675 |
|  |  | 7 | IVM | 311 ± 656 | 0-1785 | 298 ± 598 | 0-1650 |
|  |  | 7 | CON | 703 ± 947 | 15-2730 | 512 ± 517 | 0-1515 |
| 2 | NSW | 11 | MON | 164 ± 135 | 0-345 | 5 ± 10 | 0-30 |
|  |  | 11 | CLO | 217 ± 173 | 0-570 | 147 ± 146 | 0-495 |
|  |  | 11 | FBZ | 374 ± 485 | 0-1605 | 513 ± 572 | 0-1605 |
|  |  | 11 | IVM | 559 ± 983 | 60-3525 | 1372 ± 2100 | 165-7470 |
|  |  | 10 | CON | 426 ± 426 | 15-1290 | 320 ± 213 | 15-600 |
| 3 | NSW | 8 | MON | 653 ± 1027 | 0-2745 | 6 ± 11 | 0-30 |
|  |  | 8 | QDR | 1174 ± 2575 | 0-7410 | 2 ± 5 | 0-15 |
|  |  | 8 | CLO | 298 ± 462 | 0-1425 | 56 ± 96 | 0-285 |
|  |  | 8 | IVM | 108 ± 97 | 0-240 | 236 ± 377 | 0-1155 |
|  |  | 8 | CON | 204 ± 217 | 0-675 | 393 ± 560 | 0-1590 |
| 4 | WA | 14 | MON | 931 ± 3093 | 0-11670 | 8 ± 28 | 0-105 |
|  |  | 13 | QDR | 1068 ± 2052 | 0-7065 | 3 ± 9 | 0-30 |
|  |  | 14 | CLO | 205 ± 200 | 0-735 | 46 ± 84 | 0-300 |
|  |  | 14 | FBZ | 378 ± 575 | 0-1935 | 100 ± 1 | 0-615 |
|  |  | 14 | IVM | 470 ± 841 | 0-3210 | 338 ± 832 | 0-3135 |
|  |  | 15 | CON | 1619 ± 3989 | 0-735 | 618 ± 1312 | 0-300 |
| 5 | NSW | 10 | MON | 743 ± 931 | 15-2430 | 0 | 0 |
|  |  | 10 | QDR | 1349 ± 1539 | 120-5160 | 0 | 0 |
|  |  | 10 | CLO | 335 ± 443 | 30-1515 | 63 ± 86 | 0-225 |
|  |  | 10 | FBZ | 726 ± 1193 | 0-3315 | 327 ± 462 | 0-1335 |
|  |  | 10 | IVM | 1079 ± 1480 | 0-4575 | 1185 ± 2126 | 0-6570 |
|  |  | 10 | CON | 618 ± 547 | 0-1275 | 749 ± 824 | 0-2220 |
| 6 | VIC | 10 | MON | 366 ± 456 | 0-1425 | 11 ± 20 | 0-60 |
|  |  | 11 | QDR | 345 ± 516 | 0-1635 | 0 | 0 |
|  |  | 10 | CLO | 408 ± 578 | 0-1725 | 152 ± 205 | 0-540 |
|  |  | 10 | FBZ | 300 ± 793 | 0-2550 | 113 ± 257 | 0-795 |
|  |  | 10 | IVM | 327 ± 268 | 0-915 | 266 ± 249 | 30-870 |
|  |  | 10 | CON | 179 ± 286 | 0-855 | 203 ± 276 | 0-870 |
| 7 | VIC | 12 | MON | 146 ± 144 | 3 ± 9 | 0-495 | 0-30 |
|  |  | 10 | QDR | 341 ± 484 | 0 | 30-1650 | 0 |
|  |  | 10 | CLO | 102 ± 127 | 26 ± 39 | 15-345 | 0-105 |
|  |  | 10 | FBZ | 162 ± 208 | 117 ± 87 | 0-630 | 0-255 |
|  |  | 10 | IVM | 496 ± 937 | 101 ± 136 | 0-3075 | 0-390 |
|  |  | 10 | CON | 81 ± 110 | 128 ± 241 | 0-330 | 0-780 |
| 8 | VIC | 12 | MON | 236 ± 145 | 0-570 | 0 | 0 |
|  |  | 10 | QDR | 351 ± 577 | 0-1935 | 0 | 0 |
|  |  | 10 | CLO | 228 ± 145 | 0-465 | 186 ± 143 | 0-480 |
|  |  | 10 | FBZ | 260 ± 174 | 0-615 | 120 ± 102 | 0-330 |
|  |  | 10 | IVM | 239 ± 223 | 0-690 | 36 ± 65 | 0-210 |
|  |  | 10 | CON | 279 ± 274 | 0-885 | 284 ± 203 | 0-720 |
| 9 | NSW | 10 | MON | 327 ± 348 | 0-1050 | 0 | 0 |
|  |  | 10 | QDR | 498 ± 938 | 0-3075 | 0 | 0 |
|  |  | 10 | CLO | 504 ± 777 | 0-1995 | 294 ± 645 | 0-1890 |
|  |  | 10 | FBZ | 1126 ± 1400 | 0-4500 | 1108 ± 2026 | 0-5970 |
|  |  | 10 | IVM | 364 ± 493 | 0-1665 | 190 ± 316 | 0-1050 |
|  |  | 9 | CON | 262 ± 508 | 0-1545 | 178 ± 393 | 0-1215 |
| 10 | SA | 10 | MON | 1988 ± 4927 | 30-15915 | 14 ± 43 | 0-135 |
|  |  | 10 | QDR | 554 ± 695 | 90-2235 | 4 ± 14 | 0-45 |
|  |  | 10 | CLO | 1194 ± 1857 | 120-6375 | 342 ± 168 | 120-600 |
|  |  | 10 | FBZ | 268 ± 186 | 45-540 | 219 ± 196 | 0-585 |
|  |  | 10 | IVM | 848 ± 1075 | 40-3360 | 476 ± 855 | 15-2850 |
|  |  | 10 | CYD | 1506 ± 3531 | 0-11520 | 297 ± 850 | 0-2715 |
|  |  | 10 | CON | 548 ± 596 | 90-1710 | 483 ± 456 | 60-1155 |
| 11 | VIC | 10 | MON | 738 ± 1634 | 30-5265 | 0 | 0 |
|  |  | 10 | QDR | 200 ± 351 | 0-1170 | 10 ± 30 | 0-90 |
|  |  | 10 | CLO | 407 ± 378 | 30-1155 | 92 ± 107 | 0-285 |
|  |  | 9 | FBZ | 518 ± 686 | 15-1680 | 231 ± 340 | 0-975 |
|  |  | 10 | CYD | 254 ± 174 | 45-510 | 20 ± 43 | 0-140 |
|  |  | 9 | CON | 384 ± 406 | 45-1065 | 270 ± 227 | 30-720 |
| 12 | VIC | 10 | MON | 256 ± 359 | 0-1050 | 0 | 0 |
|  |  | 10 | QDR | 296 ± 333 | 0-1080 | 0 | 0 |
|  |  | 10 | CLO | 1274 ± 2393 | 75-7890 | 32 ± 37 | 0-120 |
|  |  | 10 | FBZ | 369 ± 682 | 30-2265 | 228 ± 362 | 0-1170 |
|  |  | 10 | CYD | 465 ± 955 | 0-3120 | 20 ± 36 | 0-105 |
|  |  | 10 | CON | 620 ± 1263 | 0-3945 | 914 ± 1843 | 60-5445 |
| 13 | WA | 8 | MON | 731 ± 967 | 0-2415 | 0 | 0 |
|  |  | 8 | QDR | 662 ± 1047 | 0-2880 | 4 ± 11 | 0-30 |
|  |  | 8 | CLO | 182 ± 169 | 30-495 | 45 ± 80 | 0-195 |
|  |  | 8 | FBZ | 159 ± 186 | 60-615 | 24 ± 28 | 0-75 |
|  |  | 7 | CYD | 118 ± 230 | 0-630 | 6 ± 17 | 0-45 |
|  |  | 8 | CON | 246 ± 334 | 0-990 | 433 ± 422 | 0-1095 |
| 14 | QLD | 10 | MON | 240 ± 432 | 0-1440 | 0 | 0 |
|  |  | 10 | QDR | 452 ± 1039 | 0-3315 | 2 ± 5 | 0-15 |
|  |  | 10 | CLO | 604 ± 1031 | 0-3435 | 6 ± 14 | 0-45 |
|  |  | 10 | FBZ | 81 ± 140 | 0-450 | 358 ± 728 | 0-2400 |
|  |  | 10 | CYD | 861 ± 2562 | 0-8145 | 2 ± 5 | 0-15 |
|  |  | 10 | CON | 78 ± 69 | 0-165 | 118 ± 142 | 0-420 |
| 15 | TAS | 10 | MON | 81 ± 129 | 0-360 | 0 | 0 |
|  |  | 10 | QDR | 126 ± 189 | 0-570 | 0 | 0 |
|  |  | 10 | CLO | 111 ± 209 | 0-675 | 54 ± 96 | 0-315 |
|  |  | 10 | FBZ | 122 ± 150 | 15-510 | 32 ± 37 | 0-105 |
|  |  | 10 | CYD | 22 ± 29 | 0-75 | 0 | 0 |
|  |  | 10 | CON | 184 ± 187 | 0-630 | 106 ± 119 | 0-345 |
| 16 | NSW | 10 | MON | 130 ± 175 | 0-525 | 0 | 0 |
|  |  | 10 | QDR | 300 ± 288 | 0-855 | 16 ± 47 | 0-150 |
|  |  | 10 | CLO | 243 ± 297 | 0-930 | 9 ± 19 | 0-45 |
|  |  | 10 | FBZ | 208 ± 186 | 0-435 | 124 ± 250 | 0-780 |
|  |  | 10 | CYD | 147 ± 165 | 0-435 | 246 ± 588 | 0-1905 |
|  |  | 10 | CON | 339 ± 504 | 0-1620 | 249 ± 395 | 0-1305 |
| 17 | VIC | 10 | MON | 83 ± 118 | 0-315 | 0 | 0 |
|  |  | 9 | QDR | 55 ± 70 | 0-165 | 0 | 0 |
|  |  | 9 | CLO | 143 ± 208 | 0-640 | 176 ± 323 | 0-945 |
|  |  | 9 | FBZ | 94 ± 93 | 0-225 | 54 ± 45 | 0-120 |
|  |  | 9 | CYD | 268 ± 480 | 0-1440 | 7 ± 20 | 0-60 |
|  |  | 9 | CON | 84 ± 163 | 0-480 | 60 ± 56 | 0-150 |
| 18 | NSW | 7 | MON | 1589 ± 2127 | 90-5840 | 0 | 0 |
|  |  | 10 | QDR | 658 ± 437 | 200-1640 | 0 | 0 |
|  |  | 10 | CLO | 1000 ± 1156 | 0-3800 | 44 ± 133 | 0-400 |
|  |  | 10 | FBZ | 658 ± 588 | 0-1632 | 256 ± 301 | 0-816 |
|  |  | 10 | CYD | 1846 ± 3844 | 20-12280 | 114 ± 232 | 0-672 |
|  |  | 10 | CON | 1406 ± 3623 | 0-11700 | 1178 ± 2781 | 0-9048 |
| 19 | VIC | 10 | MON | 124 ± 137 | 0-450 | 2 ± 5 | 0-15 |
|  |  | 10 | QDR | 348 ± 265 | 105-855 | 2 ± 5 | 0-15 |
|  |  | 10 | CLO | 622 ± 508 | 135-1440 | 118 ± 152 | 0-465 |
|  |  | 10 | FBZ | 412 ± 422 | 0-1065 | 258 ± 343 | 0-1080 |
|  |  | 10 | CYD | 218 ± 185 | 15-495 | 0 | 0 |
|  |  | 10 | CON | 369 ± 548 | 15-1755 | 104 ± 160 | 0-520 |
| 20 | VIC | 5 | MON | 312 ± 150 | 195-540 | 0 | 0 |
|  |  | 5 | QDR | 760 ± 930 | 90-2360 | 0 | 0 |
|  |  | 6 | CLO | 378 ± 499 | 30-1350 | 318 ± 614 | 0-1560 |
|  |  | 5 | FBZ | 312 ± 698 | 0-1560 | 191 ± 256 | 0-570 |
|  |  | 6 | CYD | 842 ± 1161 | 0-2820 | 0 | 0 |
|  |  | 5 | CON | 45 ± 92 | 0-210 | 162 ± 354 | 0-795 |

*MON- monepantel, QDR- Q-drench (contains levamisole, closantel, albendazole, abamectin), CLO- closantel, FBZ- fenbendazole, IMV- ivermectin, CYD- Cydectin (moxidectin), CON- control

NSW, New South Wales; QLD, Queensland; SA, South Australia; TAS, Tasmania; VIC, Victoria; WA, Western Australia

**Additional file 1:** **Table S2** Effect of different anthelmintics on the common gastrointestinal nematodes before and after treatment in naturally infected alpacas on 20 alpaca farms in Australia

| Farm No. | Anthelmintic | Treatment | Camelostrongylus | Cooperia | Haemonchus | Oesophagostomum | Ostertagia | Teladorsagia | Trichostrongylus |
| --- | --- | --- | --- | --- | --- | --- | --- | --- | --- |
| 1 | MON | Pre | NT | NT | NT | NT | NT | NT | NT |
|  |  | Post | 0 | 0 | 0 | 0 | 0 | 0 | 0 |
|  | CLO | Pre | NT | NT | NT | NT | NT | NT | NT |
|  |  | Post | 1 | 0 | 1 | 0 | 1 | 0 | 1 |
|  | IVM | Pre | NT | NT | NT | NT | NT | NT | NT |
|  |  | Post | 0 | 0 | 1 | 0 | 0 | 0 | 0 |
|  | FBZ | Pre | NT | NT | NT | NT | NT | NT | NT |
|  |  | Post | 0 | 0 | 1 | 0 | 0 | 0 | 0 |
|  | CON | Pre | NT | NT | NT | NT | NT | NT | NT |
|  |  | Post | 0 | 0 | 1 | 0 | 0 | 0 | 1 |
| 2 | MON | Pre | 0 | 0 | 1 | 0 | 0 | 0 | 0 |
|  |  | Post | 0 | 0 | 0 | 0 | 0 | 0 | 1 |
|  | CLO | Pre | 1 | 0 | 1 | 0 | 1 | 0 | 1 |
|  |  | Post | 1 | 1 | 0 | 0 | 1 | 0 | 1 |
|  | IVM | Pre | 1 | 0 | 1 | 0 | 1 | 0 | 1 |
|  |  | Post | 0 | 1 | 1 | 0 | 0 | 0 | 1 |
|  | FBZ | Pre | 1 | 0 | 1 | 0 | 1 | 0 | 1 |
|  |  | Post | 0 | 1 | 1 | 0 | 0 | 0 | 1 |
|  | CON | Pre | 0 | 0 | 1 | 0 | 0 | 0 | 1 |
|  |  | Post | 1 | 1 | 1 | 0 | 0 | 0 | 1 |
| 3 | MON | Pre | 1 | 0 | 1 | 0 | 1 | 0 | 0 |
|  |  | Post | 0 | 0 | 0 | 0 | 0 | 0 | 0 |
|  | QDR | Pre | 1 | 0 | 1 | 0 | 1 | 0 | 1 |
|  |  | Post | 0 | 0 | 0 | 0 | 0 | 0 | 0 |
|  | CLO | Pre | 0 | 0 | 1 | 0 | 0 | 0 | 0 |
|  |  | Post | 1 | 0 | 1 | 0 | 1 | 0 | 1 |
|  | IVM | Pre | 1 | 0 | 1 | 0 | 0 | 0 | 1 |
|  |  | Post | 0 | 0 | 1 | 0 | 0 | 0 | 0 |
|  | CON | Pre | 1 | 0 | 1 | 0 | 0 | 0 | 1 |
|  |  | Post | 1 | 0 | 1 | 0 | 1 | 0 | 1 |
| 4 | MON | Pre | 0 | 0 | 0 | 0 | 0 | 0 | 0 |
|  |  | Post | 0 | 0 | 0 | 0 | 0 | 0 | 1 |
|  | QDR | Pre | 0 | 0 | 1 | 0 | 0 | 0 | 0 |
|  |  | Post | 0 | 0 | 0 | 0 | 0 | 0 | 0 |
|  | CLO | Pre | 1 | 1 | 1 | 0 | 0 | 0 | 1 |
|  |  | Post | 0 | 0 | 0 | 0 | 0 | 0 | 0 |
|  | FBZ | Pre | 0 | 0 | 1 | 0 | 0 | 0 | 1 |
|  |  | Post | 1 | 0 | 1 | 0 | 1 | 0 | 1 |
|  | IVM | Pre | 0 | 1 | 1 | 0 | 1 | 0 | 0 |
|  |  | Post | 0 | 0 | 1 | 0 | 0 | 0 | 1 |
|  | CON | Pre | 1 | 1 | 1 | 0 | 1 | 0 | 1 |
|  |  | Post | 1 | 0 | 1 | 0 | 1 | 0 | 1 |
| 5 | MON | Pre | 1 | 1 | 1 | 0 | 1 | 0 | 1 |
|  |  | Post | 1 | 0 | 1 | 0 | 1 | 0 | 1 |
|  | QDR | Pre | 1 | 1 | 1 | 0 | 1 | 0 | 1 |
|  |  | Post | 1 | 0 | 1 | 0 | 1 | 0 | 1 |
|  | CLO | Pre | 1 | 0 | 1 | 0 | 0 | 0 | 1 |
|  |  | Post | 1 | 0 | 1 | 0 | 1 | 0 | 1 |
|  | FBZ | Pre | 0 | 1 | 1 | 0 | 0 | 0 | 1 |
|  |  | Post | 1 | 0 | 1 | 0 | 1 | 0 | 1 |
|  | IVM | Pre | 1 | 1 | 1 | 0 | 1 | 0 | 1 |
|  |  | Post | 1 | 1 | 1 | 0 | 1 | 0 | 1 |
|  | CON | Pre | 1 | 1 | 1 | 0 | 1 | 0 | 1 |
|  |  | Post | 1 | 0 | 1 | 0 | 0 | 0 | 1 |
| 6 | MON | Pre | 1 | 1 | 1 | 1 | 1 | 0 | 1 |
|  |  | Post | 1 | 0 | 0 | 0 | 1 | 0 | 0 |
|  | QDR | Pre | 1 | 1 | 1 | 0 | 1 | 0 | 1 |
|  |  | Post | 0 | 0 | 0 | 0 | 0 | 0 | 0 |
|  | CLO | Pre | 1 | 1 | 1 | 0 | 1 | 0 | 1 |
|  |  | Post | 1 | 1 | 0 | 0 | 0 | 0 | 1 |
|  | FBZ | Pre | 1 | 1 | 1 | 0 | 1 | 0 | 1 |
|  |  | Post | 1 | 0 | 1 | 0 | 1 | 0 | 1 |
|  | IVM | Pre | 1 | 1 | 1 | 0 | 1 | 0 | 1 |
|  |  | Post | 0 | 0 | 1 | 0 | 0 | 0 | 0 |
|  | CON | Pre | 1 | 1 | 1 | 0 | 1 | 0 | 1 |
|  |  | Post | 1 | 0 | 1 | 0 | 1 | 0 | 1 |
| 7 | MON | Pre | 1 | 1 | 1 | 0 | 0 | 0 | 1 |
|  |  | Post | 0 | 0 | 0 | 0 | 0 | 0 | 0 |
|  | QDR | Pre | 0 | 0 | 1 | 1 | 0 | 0 | 1 |
|  |  | Post | 0 | 0 | 0 | 0 | 0 | 0 | 0 |
|  | CLO | Pre | 0 | 0 | 1 | 0 | 0 | 0 | 1 |
|  |  | Post | 0 | 0 | 0 | 0 | 0 | 1 | 1 |
|  | FBZ | Pre | 1 | 0 | 1 | 0 | 0 | 0 | 1 |
|  |  | Post | 0 | 0 | 0 | 0 | 0 | 0 | 0 |
|  | IVM | Pre | 0 | 1 | 1 | 1 | 0 | 0 | 1 |
|  |  | Post | 0 | 0 | 1 | 0 | 0 | 0 | 0 |
|  | CON | Pre | 0 | 0 | 1 | 0 | 0 | 0 | 1 |
|  |  | Post | 1 | 1 | 1 | 0 | 0 | 0 | 1 |
| 8 | MON | Pre | 1 | 0 | 0 | 0 | 1 | 0 | 1 |
|  |  | Post | 0 | 0 | 0 | 0 | 0 | 0 | 0 |
|  | QDR | Pre | 1 | 1 | 1 | 0 | 1 | 0 | 1 |
|  |  | Post | 0 | 0 | 0 | 0 | 0 | 0 | 0 |
|  | CLO | Pre | 1 | 1 | 1 | 0 | 1 | 0 | 1 |
|  |  | Post | 1 | 1 | 0 | 0 | 1 | 0 | 1 |
|  | FBZ | Pre | 0 | 0 | 0 | 0 | 0 | 0 | 0 |
|  |  | Post | 1 | 0 | 1 | 0 | 1 | 0 | 1 |
|  | IVM | Pre | 1 | 1 | 0 | 1 | 0 | 0 | 1 |
|  |  | Post | 0 | 0 | 1 | 0 | 0 | 0 | 0 |
|  | CON | Pre | 1 | 0 | 1 | 1 | 1 | 0 | 1 |
|  |  | Post | 1 | 1 | 1 | 1 | 1 | 0 | 1 |
| 9 | MON | Pre | 0 | 1 | 1 | 0 | 0 | 0 | 1 |
|  |  | Post | 0 | 0 | 0 | 0 | 0 | 0 | 0 |
|  | QDR | Pre | 0 | 1 | 1 | 0 | 0 | 0 | 0 |
|  |  | Post | 0 | 0 | 0 | 0 | 0 | 0 | 0 |
|  | CLO | Pre | 0 | 1 | 1 | 0 | 1 | 0 | 1 |
|  |  | Post | 0 | 1 | 0 | 0 | 0 | 0 | 0 |
|  | FBZ | Pre | 0 | 1 | 1 | 0 | 0 | 0 | 0 |
|  |  | Post | 0 | 0 | 1 | 0 | 0 | 0 | 0 |
|  | IVM | Pre | 0 | 1 | 1 | 0 | 0 | 0 | 0 |
|  |  | Post | 0 | 1 | 1 | 0 | 0 | 0 | 0 |
|  | CON | Pre | 0 | 1 | 1 | 0 | 0 | 0 | 0 |
|  |  | Post | 0 | 1 | 1 | 0 | 0 | 0 | 0 |
| 10 | MON | pre | 1 | 0 | 1 | 0 | 1 | 0 | 1 |
|  |  | Post | 0 | 0 | 1 | 0 | 0 | 0 | 0 |
|  | QDR | pre | 1 | 0 | 1 | 0 | 1 | 0 | 1 |
|  |  | Post | 0 | 0 | 0 | 0 | 0 | 0 | 0 |
|  | CLO | pre | 1 | 0 | 1 | 0 | 1 | 0 | 1 |
|  |  | Post | 1 | 0 | 1 | 0 | 1 | 0 | 1 |
|  | FBZ | pre | 1 | 0 | 1 | 0 | 1 | 0 | 1 |
|  |  | Post | 1 | 0 | 1 | 0 | 1 | 0 | 1 |
|  | IVM | pre | 1 | 0 | 1 | 0 | 1 | 0 | 1 |
|  |  | Post | 1 | 0 | 1 | 0 | 1 | 0 | 1 |
|  | CYD | pre | 1 | 0 | 1 | 0 | 1 | 0 | 1 |
|  |  | Post | 0 | 0 | 1 | 0 | 0 | 0 | 1 |
|  | CON | pre | 1 | 0 | 1 | 0 | 1 | 0 | 1 |
|  |  | Post | 1 | 0 | 1 | 0 | 1 | 0 | 1 |
| 11 | MON | Pre | 1 | 0 | 1 | 0 | 1 | 0 | 1 |
|  |  | Post | 0 | 0 | 0 | 0 | 0 | 0 | 0 |
|  | QDR | Pre | 1 | 0 | 1 | 0 | 1 | 0 | 1 |
|  |  | Post | 0 | 0 | 0 | 0 | 0 | 0 | 0 |
|  | CLO | Pre | 1 | 0 | 1 | 0 | 1 | 0 | 1 |
|  |  | Post | 1 | 1 | 1 | 0 | 1 | 0 | 1 |
|  | FBZ | Pre | 1 | 0 | 1 | 0 | 1 | 0 | 1 |
|  |  | Post | 1 | 0 | 1 | 0 | 1 | 0 | 1 |
|  | CYD | Pre | 1 | 0 | 1 | 0 | 1 | 0 | 1 |
|  |  | Post | 0 | 0 | 1 | 0 | 0 | 0 | 0 |
|  | CON | Pre | 1 | 0 | 1 | 0 | 0 | 0 | 1 |
|  |  | Post | 1 | 0 | 1 | 0 | 1 | 0 | 0 |
| 12 | MON | Pre | 1 | 0 | 1 | 0 | 1 | 0 | 1 |
|  |  | Post | 0 | 0 | 0 | 0 | 0 | 0 | 0 |
|  | QDR | Pre | 1 | 1 | 1 | 0 | 1 | 0 | 0 |
|  |  | Post | 0 | 0 | 0 | 0 | 0 | 0 | 0 |
|  | CLO | Pre | 1 | 1 | 1 | 0 | 1 | 0 | 1 |
|  |  | Post | 1 | 1 | 1 | 0 | 1 | 0 | 0 |
|  | FBZ | Pre | 1 | 1 | 1 | 0 | 1 | 0 | 0 |
|  |  | Post | 1 | 0 | 1 | 0 | 1 | 0 | 1 |
|  | CYD | Pre | 1 | 1 | 1 | 0 | 1 | 0 | 0 |
|  |  | Post | 0 | 0 | 1 | 0 | 0 | 0 | 0 |
|  | CON | Pre | 1 | 0 | 1 | 0 | 1 | 0 | 1 |
|  |  | Post | 1 | 0 | 1 | 0 | 1 | 0 | 0 |
| 13 | MON | Pre | 1 | 0 | 1 | 0 | 1 | 0 | 1 |
|  |  | Post | 0 | 0 | 0 | 0 | 0 | 0 | 0 |
|  | QDR | Pre | 1 | 0 | 1 | 0 | 1 | 0 | 1 |
|  |  | Post | 0 | 0 | 0 | 0 | 0 | 0 | 0 |
|  | CLO | Pre | 1 | 0 | 1 | 0 | 1 | 0 | 1 |
|  |  | Post | 0 | 0 | 1 | 0 | 0 | 0 | 0 |
|  | FBZ | Pre | 1 | 0 | 1 | 0 | 1 | 0 | 1 |
|  |  | Post | 0 | 0 | 1 | 0 | 0 | 0 | 1 |
|  | CYD | Pre | 1 | 0 | 1 | 0 | 0 | 0 | 1 |
|  |  | Post | 0 | 0 | 0 | 0 | 0 | 0 | 0 |
|  | CON | Pre | 0 | 0 | 1 | 0 | 0 | 0 | 0 |
|  |  | Post | 0 | 0 | 1 | 0 | 0 | 0 | 1 |
| 14 | MON | Pre | 0 | 1 | 1 | 0 | 0 | 0 | 1 |
|  |  | Post | 0 | 0 | 1 | 0 | 0 | 0 | 0 |
|  | QDR | Pre | 0 | 0 | 1 | 0 | 0 | 0 | 0 |
|  |  | Post | 0 | 0 | 1 | 0 | 0 | 0 | 0 |
|  | CLO | Pre | 0 | 0 | 1 | 0 | 0 | 0 | 1 |
|  |  | Post | 0 | 0 | 0 | 0 | 0 | 0 | 1 |
|  | FBZ | Pre | 0 | 1 | 1 | 0 | 0 | 0 | 1 |
|  |  | Post | 1 | 0 | 1 | 0 | 1 | 0 | 1 |
|  | CYD | Pre | 0 | 0 | 1 | 0 | 0 | 0 | 0 |
|  |  | Post | 0 | 0 | 0 | 0 | 0 | 0 | 1 |
|  | CON | Pre | 0 | 0 | 1 | 0 | 0 | 0 | 0 |
|  |  | Post | 1 | 1 | 1 | 0 | 1 | 0 | 1 |
| 15 | MON | Pre | 1 | 0 | 0 | 0 | 1 | 0 | 1 |
|  |  | Post | 0 | 0 | 0 | 0 | 0 | 0 | 0 |
|  | QDR | Pre | 1 | 0 | 1 | 0 | 1 | 0 | 1 |
|  |  | Post | 0 | 0 | 0 | 0 | 0 | 0 | 0 |
|  | CLO | Pre | 1 | 0 | 0 | 0 | 1 | 0 | 1 |
|  |  | Post | 1 | 0 | 0 | 0 | 0 | 0 | 0 |
|  | FBZ | Pre | 1 | 0 | 1 | 0 | 1 | 0 | 0 |
|  |  | Post | 0 | 0 | 0 | 0 | 0 | 0 | 0 |
|  | CYD | Pre | 1 | 0 | 0 | 0 | 1 | 0 | 1 |
|  |  | Post | 0 | 0 | 0 | 0 | 0 | 0 | 0 |
|  | CON | Pre | 1 | 0 | 0 | 0 | 1 | 0 | 0 |
|  |  | Post | 1 | 0 | 0 | 0 | 1 | 0 | 0 |
| 16 | MON | Pre | 1 | 0 | 1 | 0 | 1 | 0 | 1 |
|  |  | Post | 0 | 0 | 0 | 0 | 0 | 0 | 0 |
|  | QDR | Pre | 1 | 0 | 1 | 0 | 0 | 0 | 1 |
|  |  | Post | 0 | 0 | 0 | 0 | 0 | 0 | 0 |
|  | FBZ | Pre | 0 | 0 | 1 | 0 | 0 | 0 | 0 |
|  |  | Post | 0 | 0 | 1 | 0 | 0 | 0 | 0 |
|  | CLO | Pre | 0 | 0 | 1 | 0 | 0 | 0 | 1 |
|  |  | Post | 0 | 0 | 0 | 0 | 0 | 0 | 0 |
|  | CYD | Pre | 0 | 0 | 1 | 0 | 0 | 0 | 0 |
|  |  | Post | 0 | 0 | 1 | 0 | 0 | 0 | 0 |
|  | CON | Pre | 0 | 0 | 1 | 0 | 0 | 0 | 0 |
|  |  | Post | 0 | 0 | 1 | 0 | 0 | 0 |  |
| 17 | MON | Pre | 1 | 1 | 1 | 0 | 1 | 0 | 1 |
|  |  | Post | 0 | 0 | 0 | 0 | 0 | 0 | 0 |
|  | QDR | Pre | 1 | 0 | 1 | 0 | 1 | 0 | 1 |
|  |  | Post | 0 | 0 | 0 | 0 | 0 | 0 | 0 |
|  | CLO | Pre | 1 | 1 | 1 | 0 | 1 | 0 | 1 |
|  |  | Post | 1 | 1 | 1 | 0 | 1 | 0 | 1 |
|  | FBZ | Pre | 1 | 1 | 1 | 0 | 1 | 0 | 0 |
|  |  | Post | 0 | 1 | 1 | 0 | 0 | 0 | 0 |
|  | CYD | Pre | 1 | 1 | 1 | 0 | 1 | 0 | 1 |
|  |  | Post | 0 | 1 | 1 | 0 | 0 | 0 | 0 |
|  | CON | Pre | 1 | 1 | 1 | 0 | 1 | 0 | 0 |
|  |  | Post | 0 | 0 | 1 | 0 | 0 | 0 | 1 |
| 18 | MON | Pre | 0 | 0 | 1 | 0 | 0 | 0 | 0 |
|  |  | Post | 0 | 0 | 0 | 0 | 0 | 0 | 0 |
|  | QDR | Pre | 1 | 0 | 1 | 0 | 1 | 0 | 1 |
|  |  | Post | 0 | 0 | 1 | 0 | 0 | 0 | 0 |
|  | CLO | Pre | 1 | 0 | 1 | 0 | 1 | 0 | 0 |
|  |  | Post | 0 | 0 | 1 | 0 | 0 | 0 | 0 |
|  | FBZ | Pre | 1 | 0 | 1 | 0 | 0 | 0 | 0 |
|  |  | Post | 0 | 0 | 1 | 0 | 0 | 0 | 0 |
|  | CYD | Pre | 1 | 0 | 1 | 0 | 1 | 0 | 1 |
|  |  | Post | 0 | 0 | 1 | 0 | 0 | 0 | 1 |
|  | CON | Pre | 1 | 0 | 1 | 0 | 1 | 0 | 0 |
|  |  | Post | 1 | 0 | 1 | 0 | 1 | 0 | 0 |
| 19 | MON | Pre | 0 | 0 | 1 | 0 | 1 | 0 | 1 |
|  |  | Post | 0 | 0 | 0 | 0 | 0 | 0 | 0 |
|  | QDR | Pre | 0 | 0 | 1 | 0 | 0 | 0 | 1 |
|  |  | Post | 0 | 0 | 0 | 0 | 0 | 0 | 0 |
|  | CLO | Pre | 0 | 1 | 1 | 0 | 1 | 0 | 1 |
|  |  | Post | 1 | 0 | 0 | 0 | 1 | 0 | 1 |
|  | FBZ | Pre | 1 | 1 | 1 | 0 | 1 | 0 | 1 |
|  |  | Post | 1 | 0 | 1 | 0 | 0 | 0 | 1 |
|  | CYD | Pre | 0 | 0 | 1 | 0 | 1 | 0 | 1 |
|  |  | Post | 0 | 0 | 0 | 0 | 0 | 0 | 0 |
|  | CON | Pre | 1 | 1 | 1 | 0 | 0 | 0 | 1 |
|  |  | Post | 1 | 0 | 1 | 0 | 1 | 0 | 1 |
| 20 | MON | Pre | 1 | 0 | 1 | 0 | 1 | 0 | 1 |
|  |  | Post | 0 | 0 | 0 | 0 | 0 | 0 | 0 |
|  | QDR | Pre | 1 | 0 | 1 | 0 | 1 | 0 | 1 |
|  |  | Post | 0 | 0 | 0 | 0 | 0 | 0 | 0 |
|  | CLO | Pre | 1 | 1 | 1 | 0 | 1 | 0 | 0 |
|  |  | Post | 1 | 0 | 0 | 0 | 1 | 0 | 1 |
|  | FBZ | Pre | 1 | 0 | 1 | 0 | 1 | 0 | 0 |
|  |  | Post | 0 | 0 | 1 | 0 | 0 | 0 | 1 |
|  | CYD | Pre | 1 | 1 | 1 | 0 | 1 | 0 | 1 |
|  |  | Post | 0 | 0 | 0 | 0 | 0 | 0 | 0 |
|  | CON | Pre | 1 | 0 | 1 | 0 | 1 | 0 | 1 |
|  |  | Post | 1 | 0 | 1 | 0 | 1 | 0 | 1 |

NT, Not -tested; 0, the nematode was not detected; 1, the nematode was detected

*MON- monepantel, QDR- Q-drench (contains levamisole, closantel, albendazole, abamectin), CLO- closantel, FBZ- fenbendazole, IMV- ivermectin, CYD- Cydectin (moxidectin), CON- control
